# Protocols for uncontrolled donation after circulatory death: a systematic review of international guidelines, practices and transplant outcomes

**DOI:** 10.1186/s13054-015-0985-7

**Published:** 2015-06-24

**Authors:** Iván Ortega-Deballon, Laura Hornby, Sam D. Shemie

**Affiliations:** Canadian National Transplant Research Program, Montréal, Canada; Research Institute McGill University Health Centre, Montréal, Canada; Centre de Prélèvement d’Organes and Laboratoire de Simulation, Hôpital du Sacré-Cœur, Montréal, Canada; Faculty of Medicine and Health Sciences, Alcalá de Henares, Madrid Spain; Helicopter Emergency Medical Service (SUMMA 112), Madrid, Spain; Critical Care Division, Montreal Children’s Hospital, Office C-806, 2300, Rue Tupper, Montreal, QC H3H 1P3 Canada; DePPaRT Study, Pediatric Critical Care, Children’s Hospital of Eastern Ontario Research Institute, Ottawa, Canada; Deceased Donation, Canadian Blood Services, Ottawa, Canada; Division of Critical Care, Montreal Children’s Hospital, McGill University Health Centre, Montreal, Canada; McGill University, Montreal, Canada

## Abstract

**Introduction:**

A chronic shortage of organs remains the main factor limiting organ transplantation. Many countries have explored the option of uncontrolled donation after circulatory death (uDCD) in order to expand the donor pool. Little is known regarding the variability of practices and outcomes between existing protocols. This systematic review addresses this knowledge gap informing policy makers, researchers, and clinicians for future protocol implementation.

**Methods:**

We searched MEDLINE, EMBASE, and Google Scholar electronic databases from 2005 to March 2015 as well as the reference lists of selected studies, abstracts, unpublished reports, personal libraries, professional organization reports, and government agency statements on uDCD. We contacted leading authors and organizations to request their protocols and guidelines. Two reviewers extracted main variables. In studies reporting transplant outcomes, we added type, quantity, quality of organs procured, and complications reported. Internal validity and the quality of the studies reporting outcomes were assessed, as were the methodological rigour and transparency in which a guideline was developed. The review was included in the international prospective register of systematic reviews (Prospero, CRD42014015258).

**Results:**

Six guidelines and 18 outcome studies were analysed. The six guidelines are based on limited evidence and major differences exist between them at each step of the uDCD process. The outcome studies report good results for kidney, liver, and lung transplantation with high discard rates for livers.

**Conclusions:**

Despite procedural, medical, economic, legal, and ethical challenges, the uDCD strategy is a viable option for increasing the organ donation pool. Variations in practice and heterogeneity of outcomes preclude a meta-analysis and prevented the linking of outcomes to specific uDCD protocols. Further standardization of protocols and outcomes is required, as is further research into the role of extracorporeal resuscitation and other novel therapies for treatment of some refractory cardiac arrest. It is essential to ensure the maintenance of trust in uDCD programs by health professionals and the public.

**Electronic supplementary material:**

The online version of this article (doi:10.1186/s13054-015-0985-7) contains supplementary material, which is available to authorized users.

## Introduction

A chronic shortage of organs remains the main factor limiting organ transplantation for patients with end-stage organ failure. Although organ transplants save thousands of lives and transform the quality of life of thousands more, many people will die or remain on renal replacement therapy because the organ supply falls drastically short of demand. In Europe, nearly 99,000 patients were waiting for an organ in 2013 whilst the number of deceased donors has remained stable at approximately 9900 [1]. This is also the case in the US, where 30,000 patients were on waiting lists and the number of deceased donors was 8268 [1]. In Canada, the situation is equally concerning. At the end of 2013, 4433 patients were on the waiting lists and only 553 actual deceased donors were obtained that year [2]. The mismatch between supply and demand for organs has led policy makers and health institutions to develop new strategies aimed at expanding the organ donor pool. As a result, many countries worldwide have explored the option of donation after circulatory death (DCD).

The DCD procedure seeks to obtain solid organs from patients previously declared dead following the cessation of their circulatory and respiratory functions. There are two distinct methods: controlled DCD (cDCD) and uncontrolled DCD (uDCD). The cDCD occurs after an anticipated in-hospital cardiac arrest, generally but not exclusively in intensive care unit patients who have suffered a catastrophic brain injury and for whom a decision has been made to withdraw life-sustaining therapies (WLST). In this scenario, consent for cDCD is obtained, WLST occurs and a variable amount of time later, death is declared, and organs are procured. The uDCD is initiated following an unexpected, and usually out-of-hospital, refractory cardiac arrest. After resuscitation attempts are judged futile, interventions—ongoing cardiac compressions and mechanical ventilation—are initiated to preserve organs for donation. The diagnosis of death may occur after resuscitation is terminated on scene or after arrival to the hospital. There is a “no touch” period after which death is determined and organ preservation may be restarted. After hospital arrival, cannulation and organ preservation with extracorporeal perfusion or *in situ* cooling begin. Consent requirements for donation and organ preservation vary by region and may occur before or after cannulation.

Protocols for uDCD have already been implemented in Spain, France, Italy, the UK, and The Netherlands [3]. Protocols have also been developed in other countries, such as Belgium, Switzerland, and Austria, and in Saint Petersburg (Russia) and in New York City [4]. These international experiences have demonstrated that uDCD is an effective way to increase the availability of solid organs for transplantation [5]. Although uDCD appears to have promising results in terms of graft survival, it raises several medical, ethical, legal, economic, and logistic challenges at the intersection of cardiac arrest, resuscitation, organ donation, and organ preservation after declaring death [6, 7]. Little is known regarding the variability of practices between existing protocols [8] and less still regarding the comparative effectiveness of implementing a particular protocol [9].

The purpose of this systematic review is to address this knowledge gap by compiling and analyzing the defining elements and reported transplant outcomes of the currently active protocols and guidelines for uDCD. To the best of our knowledge, no systematic review has been conducted to specifically evaluate and compare the practices and outcomes of uDCD protocols, nor has any evaluation of the quality of guidelines for implementing such protocols been performed. This review will inform uDCD protocol and practice development, which has applicability to policy makers, researchers, and clinicians to assist in future protocol implementation.

## Methods

### Design of the study and search strategy

We conducted a systematic review of the literature in accordance with reviews in health care from the Center for Reviews and Dissemination, from the University of York [10]. We used a modified PICOTS format: Population: potential uDCD candidates; Intervention: active protocols for uDCD; Control: not applicable; Outcomes: in terms of (a) define elements of international practices on protocols for uDCD and, when reported (b) grafts obtained or transplanted (or both), as well as graft or patient survival and complications (or both); Time: 2005 to March 2015; and Setting: any organization that has produced a recommendation or protocol for uDCD.

We developed a comprehensive search strategy with the help of a qualified librarian. We searched MEDLINE, EMBASE, and Google Scholar electronic databases from 2005 to March 2015. The search included English, French, Italian, and Spanish and was limited to human studies. We manually searched the reference lists of selected studies and the grey literature for unpublished reports, personal libraries, professional organization, and government agency statements on uDCD. We also contacted leading authors and organizations in the field of uDCD to request their protocols and guidelines.

### Eligibility criteria

Our inclusion criteria for review were any kind of report proposing a clinical procedure for uDCD endorsed by a government agency, professional organization, professional society, or regional health-care organization. We excluded any editorials, letters, abstracts, or personal opinion articles that were not supported by the aforementioned organizations.

### Study selection

**Fig. 1:**
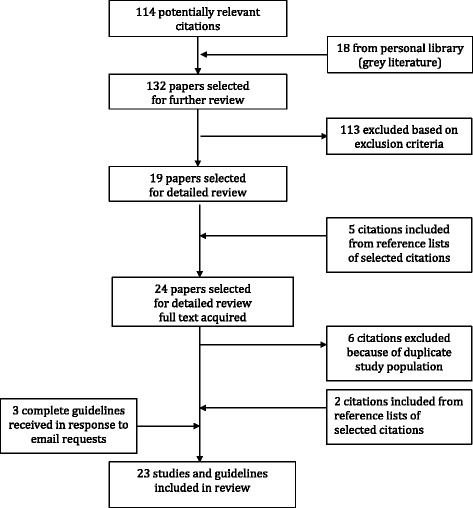
Flow chart of study selection process

Two trained reviewers (IO-D and LH) screened all citations. We retrieved the full texts of selected citations and independently reviewed them to assess study eligibility. Disagreements were resolved by consensus or with the intervention of a third expert reviewer (SDS). We used EndNote manager software (EndNote X7.1 version by Thomson Reuters) to manage the collection of publications. Figure 1 describes the study selection process.

### Data extraction and quality assessment

Two reviewers (IO-D and LH) extracted data. We created an Excel (Excel version 2013 by Microsoft Office, Microsoft Corporation, Redmond, WA, USA) data collection tool that was piloted in a sample from the list of included studies. The final version of the spreadsheet included the following variables: name of the authors, country, language, setting, year, type of study and method, eligibility criteria for population, intervention and timelines during process, organ preservation details, death determination characteristics, type and time of consent, and any ethical, legal, and logistic issues described. For the studies reporting transplant outcomes, we added type, quantity, quality of organs procured, and complications reported. Internal validity of the studies was assessed independently by two reviewers (IO-D and LH). The quality of the studies reporting outcomes was assessed by using the Downs and Black scale [11]. The Appraisal of Guidelines for Research and Evaluation (AGREE) Instrument version II [12] was used to assess the methodological rigour and transparency of guidelines. Up to three reviewers assessed each guideline.

### Data synthesis

We anticipated heterogeneity in selected studies and guidelines. Variability was apparent in eligibility criteria, organs obtained, timelines along the ischaemia process, determination of circulatory death practices, ischaemia definition, and techniques for organ preservation. Therefore, pooling of study data was not feasible and a meta-analysis was not possible. Rather, data analysis consisted of a tabulation of characteristics from studies and guidelines.

### Review end points

This systematic review aimed to address the following question: What are the defining elements and reported outcomes of currently active protocols and recommendations for uDCD?

## Results

After launching the search strategy (Fig. 1), we obtained 114 potentially relevant citations, in addition to 18 from grey literature, resulting in a total of 132 references for further review. Of these, 113 were excluded after a first screening for the following reasons: not relevant (*n* = 42); editorials, surveys, or opinions (*n* = 32); referring to results from cDCD (*n* = 19); duplicated data (*n* = 16); and case reports or abstracts (*n* = 4). The resulting 19 references were selected for further review, and five new citations were included from their reference lists. Therefore, a total of 24 references were screened after acquiring the full-text version. Following the full-text review, six citations were excluded because the study population was duplicated while two other citations from reference lists were included. During the second screening, we contacted agencies of different countries involved in implementation of protocols for uDCD and received three more guidelines in response to our request. Thus, a final total of 23 references—17 studies [13–29], five guidelines [4, 30–33], and one article [34] that included both a full guideline description and transplant outcomes—was included. Thus, for the purpose of this review, six guidelines and 18 outcome studies were analysed.

### Main characteristics of guidelines

**Fig. 2:**
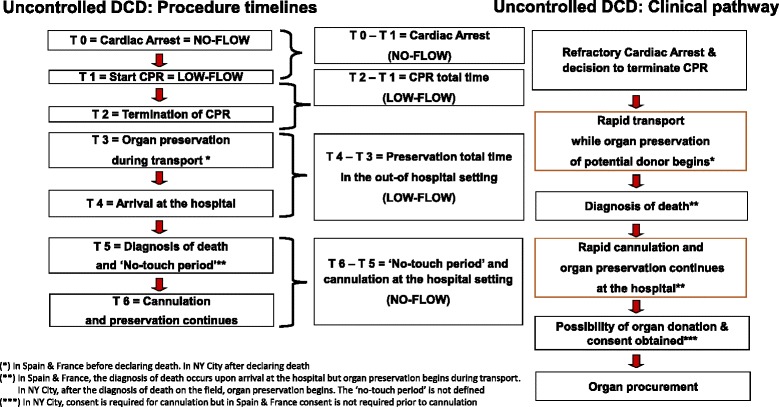
Timelines and clinical pathway in the process of uncontrolled donation after circulatory death (DCD). *CPR* cardiopulmonary resuscitation

Figure 2 is an illustrative example of the uDCD procedure timelines and clinical pathway described within the guidelines. Timelines begin with a cardiac arrest, followed by initiation of cardiopulmonary resuscitation (CPR), termination of CPR, continuation of organ-preserving interventions, diagnosis of death, and cannulation for organ preservation. As will be further described below, there are variable periods of no-flow and low-flow states that may impact on pre- and post-mortem ischemic organ injury and there is variability in the timing of, and requirement for consent for, donation or organ preservation or both.

In our review, we included six guidelines from as many countries. The main characteristics are described in Table 1 (“Guidelines” section) and summarized here.

**Table 1:** Characteristics of included guidelines and eligible outcome studies

Guidelines (*n* = 6)				
National/Regional Guideline Country; region (year)	Language	Population targeted	Location of cardiac arrest	Organ(s)
FRANCE (2007) [30]	French	Adult	Out of hospital and in hospital	Kidney and liver
ITALY; Pavia (2011) [34]	English	Adult and children (≥15 years)	Out of hospital and in hospital	Kidney
SWITZERLAND (2011) [32]	French	Adult and children (≥16 years)	Out of hospital and in hospital	Kidney
US; New York City (2011) [4]	English	Adult and children	Out of hospital	Kidney and
				liver (phased in)
SPAIN [31];				
Alicante (2012)	Spanish	Adult and children (≥14 years)	Out of hospital	Kidney, liver, and lung
Barcelona (2012)		Adult and children (≥14 years)		Kidney and liver
Castilla La Mancha (2012)		Adult and children (≥7 years)		Kidney, liver, and lung
Granada (2012)		Adult and children (≥7 years)		Kidney, liver, and lung
Galicia (2012)		Adult and children (≥14 years)		Kidney and liver
Madrid City and Region (2012)		Adult and children		Kidney, liver, and lung
UK; Scotland (2013) [33]	English	Adult and children (≥16 years)	Out of hospital	Kidney, Liver and lungs
Eligible outcome studies (*n* = 18)				
Study/Country, Region	Study design	Population studied	Location of cardiac arrest	Organ(s)
Gámez 2005 [13]/Spain, Madrid	Case series	Adult	Out of hospital	Lung
Gagandeep 2006 [14]/USA, Nationwide	Database review comparison to DBD and cDCD	Adult and children	in hospital	Kidney
Sánchez-Fructuoso 2006 [15]/Spain, Madrid	Retrospective cohort	Adult	Out of hospital and in hospital	Kidney
Fondevila 2007 [16]/Spain, Barcelona	Retrospective cohort with Matched DBD controls	Age not specified	Out of hospital	Liver
Suárez 2008 [17]/Spain, La Coruna	Retrospective cohort compared to HBD	Adult	Out of hospital and in hospital	Liver
Fieux 2009 [18]/France, Paris	Prospective cohort	Adult	Out of hospital	Kidney
Gómez Gutiérrez 2009 [19]/Spain, La Coruña and Madrid	Case series	Adult	Out of hospital	Liver
Jiménez-Galanes 2009 [20]/Spain, Madrid	Prospective case Control matched to DBD	Adult	Out of hospital	Liver
Ribalta 2009 [29]/Spain, Cataluña	Retrospective cohort	Adult and children	Out of hospital	Kidney and liver
Mateos-Rodríguez 2010 [21]/Spain, Madrid	Retrospective cohort	Adult and children	Out of hospital	Kidney, liver, and lungs
Mateos-Rodríguez 2010 [22]/Spain, Madrid	Retrospective cohort	Adult	Out of hospital	Kidney
Geraci and Sepe 2011 [34]/Italy, Pavia	Retrospective Cohort	Adult	In hospital	Kidney
Hoogland 2011 [23]/The Netherlands, Maastricht	Retrospective cohort compared with cDCD	Adult	Out of hospital	Kidney
Rodríguez 2011 [24]/Spain, Madrid and Santander	Retrospective cohort	Adult and children	Out of hospital	Lungs
Fondevila 2012 [25]/Spain, Barcelona	Retrospective cohort	Adult	Out of Hospital	Liver
Gómez-de-Antonio 2012 [26]/Spain, Madrid	Prospective cohort	Adult	Out of hospital	Lung
Hanf 2012 [27]/France, Lyon	Prospective cohort compared with ECD and SPK	Adult	Out of hospital	Kidney
Reznick 2013 [28]/Russia, St Petersburg	Retrospective cohort	Adult	In hospital	Kidney

Extended criteria donors (ECDs) were all donors at least 60 years old and those 50–59 years old with at least two of the other three conditions (cerebrovascular cause of death, renal insufficiency with serum creatinine less than or equal to 1.5 mg/dl, and hypertension)

*DBD* donation after brain death, *cDCD* controlled donation after circulatory death, *HBD* heart beating donors, *SPK* simultaneous non-sensitized kidney pancreas transplanted patients that received kidneys from optimal donors

Please note that the article by Geraci and Sepe (2011) was included in both the “Guidelines” and “Eligible outcome studies” sections of this table because it included both a guideline for a protocol and its preliminary results

### Cardiac arrest location and uncontrolled donation after circulatory death donor definition

All of the guidelines describe potential uDCD donors as those patients suffering an out-of hospital refractory cardiac arrest after failed resuscitation in the field. The guidelines from France, Italy, and Switzerland also consider in-hospital patients as potential donors. Age limits for donors most commonly included adults and older teenagers, but children were also eligible in some regions of Spain (Table 1).

### Geographic implementation and organs procured

In the case of France and Spain, national recommendations do exist for uDCD. The uDCD strategy has been implemented in Spain since 1995 with the pioneering Madrid program, yet national recommendations were not published until 2012. In France, guidelines were published in 2007 and only after this were several uDCD programs implemented. In the case of Italy, the protocol has also achieved results but is operating only in the region of Pavia. The protocol of New York City, though running, has not reported any transplant outcomes. In Scotland, a standard operating procedure is being piloted at Edinburgh. Spanish recommendations include seven uDCD protocols operating in six different regions, including Madrid which has two programs. At present, the guidelines from both Spain and the UK include procedures for recovering kidneys, livers, and lungs. France is recovering kidneys and livers. In Italy, kidneys are being procured. Switzerland, New York City, and Scotland consider only kidney procurement. A summary of the specific details of the uDCD process contained within the guidelines is included in Table 2 (“Guidelines” section) and this process is further described here.

**Table 2:** Summary of specific details of included guidelines and eligible outcome studies

	Death declaration		Time restrictions				Organ preservation					Ethical and legal issues addressed						Logistic issues	
	Definition of refractory cardiac arrest (time of CPR in min)	“No touch” time (min)	Max arrest time with no CPR (min)	Max time - CPR to cannulation (min)	Max time - cardiac arrest to cannulation (min)	Max time – cannulation to procurement (min)	Cannulation permitted prior to consent	n-ECMO used	h-ECMO used	*In situ* cooling	Pulsatile perfusion	Information given to next of kin on the field	Organ preservation initiated in ambulance during transport	Consent for cannulation of the cadaver for organ preservation	Objective of intra-aortic balloon	Health providers’ attitudes and beliefs	ECMO: organ preservation versus lifesaving technique	Cost-effectiveness evaluation	Coordination efforts needed
Guidelines (*n* = 6)																			
France (2007) [30]	30 ACLS	5	30	90 mCPR 120 aCPR	120 mCPR 150 aCPR	180 ISC 240 ECMO	N	N	Y	Y	Y	Y	Y	Y	N	Y	N	N	Y
Italy; Pavia (2011) [34]	NS	20	15	110	125	360	N	Y	N	N	Y	N	Y	Y	N	N	N	Y	Y
Switzerland (2011) [32]	20	10	30	120	150	180	Y	Y	N	Y	N	N	N	N	N	N	N	N	N
US; New York City (2011) [4]	30	NS	NS	120	NS	240	N	Y	N	N	Y	Y	Y OPV	Y	Y	Y	Y	Y	Y
Spain; Alicante, Barcelona, Castilla La Mancha, Granada, Galicia, Madrid City and Region (2012) [31]	Failed CPR	5	15 A, C, Gr, M 20 Ga 30 B	120	150	120 ISC 240–360 ECMO	Y	Y A B C Gr M	Y M	Y A Ga Gr	Y B M	Y	Y	Y	Y	Y	N	Y	y
UK; Scotland (2013) [33]	Failed CPR	5	15	105	120	NS	Y	Y	N	N	Y	N	N	Y	Y	Y	N	Y	Y
Eligible outcome studies (*n* = 18)																			
Gámez 2005 [13]/Spain, Madrid	30	5	15	105	120	240	Y	N	Y	Y	N	N	N	N	N	N	N	N	Y
Gagandeep 2006 [14]/USA, Nationwide	NS	NS	NS	NS	NS	NS	NS	NS	NS	NS	NS	NS	NS	NS	NS	NS	NS	Y	Y
Sánchez-Fructuoso 2006 [15]/Spain, Madrid	30	5	15	105	120	240	Y	N	Y	Y	Y	N	N	N	N	N	N	N	N
Fondevila 2007 [16]/Spain, Barcelona	NS	5	15	135	150	240	Y	Y	N	N	Y	N	N	Y	N	N	N	N	N
Suárez 2008 [17]/Spain, La Coruna	NS	5	15	105	120	240	Y	Y	Y	N	N	N	N	N	N	N	N	N	N
Fieux 2009 [18]/France, Paris	30 ACLS	5	30	90 mCPR 120 aCPR	120 mCPR 150 aCPR	180 ISC 240 ECMO	N	N	Y	Y	Y	Y	Y	Y	N	Y	N	N	Y
Gómez Gutierrez 2009 [19]/Spain, La Coruña and Madrid	Failed CPR	5	NS	NS	120	130	Y	Y	N	Y	N	N	N	N	N	Y	N	N	Y
Jiménez-Galanes 2009 [20]/Spain, Madrid	Failed CPR	5	15	135	150	240-270	Y	Y	N	N	NS	N	N	Y	N	N	N	N	Y
Ribalta 2009 [29]/Spain, Cataluña	Failed CPR	5	30	120	150	NS	Y	NS	NS	NS	Y	N	N	N	N	N	N	N	Y
Mateos-Rodríguez 2010 [21]/Spain, Madrid	Failed CPR	5	15	105	120	NS	Y	Y	NS	N	NS	N	N	N	N	N	Y	N	Y
Mateos-Rodríguez 2010 [22]/Spain, Madrid	30	5	15	105	120	240	Y	Y	N	NS	NS	N	N	N	N	N	Y	N	Y
Geraci and Sepe 2011 [34]/Italy, Pavia	NS	20	15	110	125	360	N	Y	N	N	Y	N	Y	Y	N	N	N	Y	Y
Hoogland 2011 [23]/The Netherlands, Maastricht	NS	5	NS	90	NS	NS	Y	N	N	Y	Y	N	N	Y	N	N	Y	N	Y
Rodríguez 2011 [24]/Spain, Madrid and Santander	NS	5	10	110	120	240	Y	N	N	Y	Y	N	N	N	N	N	N	N	N
Fondevila 2012 [25]/Spain, Barcelona	20	5	15	150	165	240	Y	Y	N	N	N	N	N	Y	N	N	N	Y	N
Gomez-de-Antonio 2012 [26]/Spain, Madrid	NS	5	15	105	120	240	Y	N	N	Y	Y after2009	N	N	N	N	N	N	N	N
Hanf 2012 [27]/France, Lyon	30	5	30	90 mCPR 120 aCPR	120 mCPR 150 aCPR	180	NS	Y	N	Y	Y	N	N	N	N	N	N	N	N
Reznick 2013 [28]/Russia, Saint Petersburg	Failed CPR	NS	60^a^	NS	NS	NS	Y	Y	N	N	NS	N	N	Y	N	N	Y	N	Y

*CPR* cardiopulmonary resuscitation, *n-ECMO* normothermic extracorporeal membrane oxygenation, *h-ECMO* hypothermic extracorporeal membrane oxygenation, *ECMO* extracorporeal membrane oxygenation, *ACLS* advanced cardiac life support, *mCPR* manual cardiopulmonary resuscitation, *aCPR* automated cardiopulmonary resuscitation, *ISC in situ* cooling, *OPV* organ preservation vehicle, *N* the procedure is not used or the issue is not discussed, *Y* the procedure is used or issue is discussed, *NS* not specified (no information specified in guideline or study), *A* Alicante, *C* Castilla La Mancha, *Gr* Granada, *M* Madrid City and Region, *Ga* Galicia, *B* Barcelona

^a^time is after failed CPR until Organ Procurement Organization arrival

### Death declaration and time restrictions

For the studies that provided a definition of refractory cardiac arrest, it is defined as 30 minutes of failed resuscitation. Death determination in France and Switzerland obliges the absence of circulation, spontaneous ventilation, and the performance of a rapid neurologic assessment to confirm the absence of consciousness, spontaneous motor activity, and brainstem reflexes. A rapid neurological testing is also performed in the New York City protocol in the prehospital setting. Scotland determines death in the emergency department after 5 minutes of absent cardiopulmonary activity defined by the absence of respiratory effort and no electrical activity on the electrocardiogram, no cardiac movement on focused echocardiography, or no pressure wave visible on the arterial line tracing. A “no-touch period”, defined as a hands-off interval, during which no interventions to the body are allowed, is required for declaring death. This period follows the decision to stop resuscitation or organ preservation attempts and varies widely between protocols, ranging from 5 to 20 minutes. There is also wide variation with respect to the maximum allowable times for each of the following periods: cardiac arrest prior to CPR (range of 15 to 30 minutes), CPR to cannulation (range of 90 to 120 minutes), and cannulation to organ procurement (range of 120 to 360 minutes).

### Organ preservation

All six guidelines recommend femoral arterial and venous cannulation and extracorporeal membrane oxygenation (ECMO) (re-initiation of circulation with an oxygenated solution). Spain recommends both normothermic and hypothermic conditions, whereas France recommends hypothermic ECMO. Scotland, Italy, and New York City use normothermic ECMO. In Spain and France, depending on center experience, the organ preservation techniques may also include *in situ* cooling with flushing of cold preservation fluids into the abdominal cavity and/or pleural spaces. Switzerland considers *in situ* cooling preservation and normothermic ECMO. The *ex vivo* renal perfusion machine is being used in Spain, France, and Italy and was proposed in Scotland and New York City. Spain has recently expanded the use of *ex vivo* organ perfusion for lung preservation in some centers.

### Ethical, legal, and logistic issues

To a variable extent, all of the included guidelines address a number of ethical, legal, and logistic issues associated with uDCD. Table 2 describes various issues in guidelines, including information provided to next of kin in the field (4/6), organ preservation initiated in the ambulance during transport (4/6), consent for cannulation and procurement (5/6), objective of inserting an intra-aortic balloon (3/6), health providers’ attitudes and beliefs (4/6), role of ECMO organ-preserving versus lifesaving technique (1/6), and cost-effectiveness considerations (4/6).

### Guideline appraisal

To assess the rigour of clinical practice guideline development, the six documents from countries with national or regional guidelines were evaluated. Additional file 1 contains an appraisal of each of the guidelines. In accordance with the AGREE II appraisal process, scaled scores for each of six different domains are presented. After a global interpretation of the quality scores, we observed that lower scores for all the guidelines assessed were in the domains of “Rigour of development”, “Applicability”, and “Editorial independence”. The higher-quality scores were obtained in the domains of “Scope and purpose” and “Clarity of presentation”. In regard to the domain of “Stakeholder involvement”, the quality scores were low or fair for all assessed guidelines, with the exception of the New York City protocol, which obtained the highest score.

### Main characteristics and protocol details of studies reporting transplant outcomes

Our review included 18 studies that reported outcomes for recipients of organs recovered by uDCD protocols. The main characteristics of the studies are described in Table 1 (“Eligible outcome studies” section) and are summarized here.

### Types of studies and organs procured

The included studies were carried out at centres in Spain, France, the US, The Netherlands, and Russia. There were no randomized controlled trials; all studies were observational in nature. Three studies were prospective cohorts [18, 26, 27], one study was a prospective case control [20], one was a retrospective cohort with matched controls [16], three were retrospective cohorts with comparisons to cDCD or donation after brain death [14, 17, 23], eight were retrospective cohorts [15, 21, 22, 24, 25, 28, 29, 34], and two were case series [13, 19]. The organs procured were kidneys (10 studies), livers (seven studies), and lungs (four studies). Although one study reported results for all three organs [21] and another for two organs [29], most studies were focused on single-organ procurement.

### Age of donors and location of cardiac arrest

Potential donors were mostly young adults (between 18 and 65 years), and only a few studies included pediatric populations [14, 21, 24, 29]. Trends to limit the upper age when liver or lungs are procured do exist [13, 16, 17, 19, 20, 24–26]. The potential donors were recruited mainly outside the hospital (OHCA) in Europe, although some studies from Spain [15, 17] and one from Italy [34] also enrolled potential donors after presenting in-hospital cardiac arrest (IHCA). Outcome studies from the US [14] and Russia [28] restricted uDCD donors to IHCA. A summary of the specific details of the uDCD process for the studies reporting outcomes is included in Table 2 (“Eligible Outcome Studies” section) and this process is further described here.

### Refractory cardiac arrest to death declaration

Based on the illustrative uDCD clinical pathway timelines in Fig. 2, permissible timelines are reviewed in Table 2. Maximum times limits were reported for each of the following intervals: cardiac arrest prior to CPR (no-flow, range of 10 to 30 minutes), CPR to cannulation (low flow, range of 90 to 150 minutes for OHCA), and cannulation to organ procurement (range of 130 to 270 minutes). There is wide variability among studies with respect to the criteria for determining when a sudden cardiac arrest is considered to be refractory to resuscitation. Most reports refer to failed CPR without defining CPR duration. In the studies in which it was specified, death determination was based only on circulatory criteria, with the exception of the two French studies [18, 27] in which an additional neurologic screening was performed according to legal requirements. In the Russian single-center experience [28], always after an IHCA, resuscitation attempts were stopped after being judged futile and then a “no-touch period”, of up to 60 minutes, occurred while waiting for the organ procurement team to arrive. Based on these findings, the so-called warm ischemic time (WIT), resulting from the addition of “no-flow” and “low-flow” periods until the beginning of in-hospital preservation techniques that were instituted, ranged from 120 to 150 minutes.

### Organ preservation

Three different organ-preserving options were described: *in situ* cooling preservation of abdominal organs or lungs and hypothermic (h-ECMO) and normothermic (n-ECMO) extracorporeal membrane oxygenation. Both h-ECMO and n-ECMO recirculate a preservation liquid and oxygenated blood through the body of the donor. The insertion of an inflated intra-aortic balloon was widely used when the ECMO technique was deployed to isolate the perfusion of abdominal organs and to avoid the reperfusion of the heart and brain [16, 17, 21, 22, 25]. A trend in the use of the *ex vivo* perfusion machine was observed [15, 16, 18, 23, 24, 29]. The time of organ preservation using the above-described techniques (so-called “cold ischemic time”) varied from 180 to 270 minutes.

### Ethical, legal, and logistic issues

Heterogeneity in practice was evidenced in terms of the requirement and timing of consent for both beginning preservation and the procurement of organs as well as for who the consent is obtained from (donor next of kin or recipient of an organ procured from uDCD donor or both). In addition, a number of ethical, legal, and logistic issues derived from daily practice were discussed by the authors. These included the type of information provided to next of kin in the field, consent requirements (if any), stated goals of intervention for pre-hospital and in-hospital organ preservation, and the potential conflict of interest between lifesaving and organ-preserving ECMO.

### Appraisal of outcome study quality

Additional file 2 contains the quality assessment of the outcome studies. All studies were observational in nature and therefore by design were generally of low quality. Most presented high risk of confounding, risk of bias, and threats to external validity.

### Transplant outcomes

We reviewed the outcomes from 10 studies procuring kidneys, seven procuring livers and four where lungs were obtained (Table 3). Larger sample sizes were derived from the Spanish experience [15, 17, 19–22, 24–26] and the two reported multicenter retrospective cohort reviews [14, 23]. Kidneys transplanted from uDCD donors demonstrated fair [14, 15, 23, 28] or poor [18, 27] early results in terms of delayed graft function, although all studies reported good results for graft and patient survival in the short and medium terms. Liver transplants from uDCD donors reported a low percentage of primary non-function and acceptable graft and patient survivals, but in all cases this was at the expense of discarding a high proportion of potential livers [16, 17, 19–21, 25, 29]. Although the experience with transplanted lungs is still limited, there are significant rates of acute rejection and primary non-function of the graft as well as medical complications [13, 24, 26]. However, short- and medium-term graft and patient survival are improving considerably based on the results of the most recent study [26]. (Complete outcome data can be found in Additional file 3).

**Table 3:** Outcomes of included studies (*n* = 18)

Outcome studies	Time period	Total donors	Total recipients	Outcomes
by organ type		n	n	
Lung – 3 studies				
• 1 case series [13]	2002 to 2009	66	67	Time to extubation: 21 hours–144 days
• 1 retrospective cohort [24]				
				Hospital stay: 20–59 days
				Primary graft dysfunction: 17–46.9 %
• 1 prospective cohort [26]				
				1-year patient survival: 68 %
				3-year patient survival 57 %
				5-year patient survival 51 %
No comparisons were made to outcomes using cDCD or DBD donors.				
Kidney – 8 studies				
• 1 database review [14]	1981 to 2011	750	629^a^	Primary graft non-function: 0–22 %
				Delayed graft function: 51–92 %
• 5 retrospective cohort [15, 22, 23, 28, 34]				
				1-year graft survival: 87.4–100 %
• 2 prospective cohort [18, 27]				
				3-year graft survival: 100 %
				5-year graft survival: 63–82.1 %
				10-year graft survival: 50 %
				1-year patient survival: 95–100 %
				3-year patient survival: 100 %
				5-year patient survival: 78–90 %
				10-year patient survival: 61 %
Three studies compared outcomes with DBD donors; two studies reported no significant differences in primary graft non-function, graft survival, and patient survival, but delayed graft function was significantly higher for recipients of uDCD kidneys.				
One study compared outcomes with cDCD donors and reported no difference in any of the outcomes.				
Liver – 5 studies				
• 1 case series [19]	1994 to 2010	122	122	Primary graft non-function: 10–18 %
• 3 retrospective cohort [16, 17, 25]				
				1-year graft survival: 50–80 %
• 1 prospective case–control [20]				5-year graft survival: 49 %
				1-year patient survival: 70–85.5 %
				5-year patient survival: 62 %
Four studies compared outcomes with DBD donors and reported no significant differences in 1-year graft and patient survival and 5-year patient survival, but primary graft non-function was significantly higher and 5-year graft survival was significantly lower for recipients of uDCD livers				
Kidney and liver – 1 study				
• 1 retrospective cohort [29]	2008	34 K 4 L	NR	No outcomes reported
Kidney, liver, and lung – 1 study				
• 1 retrospective cohort [21]	2005 to 2008	82	158 K 16 L 13 LG	Primary graft non-function of kidneys: 9 %
				Rejection rate of kidneys: 9 %
				Acute rejection rate of liver: 25 %
				No outcomes reported for lungs

*cDCD* controlled donation after circulatory of death, *DBD* donation after brain death, *uDCD* uncontrolled donation after circulatory death, *K* kidney, *L* liver, *NR* not reported, *LG* lung

^a^Gangandeep did not report number of recipients and Fieux 2009 reported outcomes for 24/31 recipients. (Complete outcome data can be found in Additional file 3)

## Discussion

uDCD is a complex and labour-intensive process. Although there has been an extended experience with uDCD in Spain, pioneering the strategy with seven programs, the international experience remains at an early stage of development and thus critical analysis and summative evaluation are difficult. The purpose of this review was to assemble and evaluate the uDCD guidelines and outcomes in order to inform the medical, ethical, legal, and logistic issues to be addressed in the ongoing development of future protocols and health policy. We summarize two sources to inform practice: international guidelines and transplant outcome reports. We have created an illustration of the clinical pathway and timelines that describe the process (Fig. 2).

Several countries in Europe (France, Italy, Scotland, Spain, and Switzerland) have guidelines for uDCD. In North America, after several failed attempts to implement this strategy [35], New York City is the only area to have developed uDCD guidelines. Assessment of uDCD guidelines by using the AGREE II appraisal process revealed that although most of the guidelines scored well in relation to the domains of “Scope and purpose”, “Stakeholder involvement”, and “Clarity of presentation”, improvements were necessary in the domains of “Rigour of development” and “Editorial independence”.

We evidenced wide variability of recommendations regarding the definitions of and time limits associated with death declaration as well as “no flow” and “low flow” periods. The practices associated with ante-mortem or post-mortem intervention, the logistic pathway, and the organ-preserving techniques used throughout process were also inconsistent.

The heterogeneity of the outcome studies prevents any meaningful comparison between programs. With these limitations in mind, it appears that uDCD can provide viable, good-quality organs. There will need to be better consistency and clarity in the reporting of outcomes, standardized definitions of each step of the ischaemia process and higher homogeneity of follow-up times for both graft and patient survival.

All of the reviewed guidelines included specific concerns with ethical, legal, and logistic implications. Many authors [5–7, 9, 22, 23, 31, 35–53] have pointed out that protocols for uDCD entail specific challenges. These issues, if unresolved, may hinder further worldwide development of uDCD strategy [9]. Specifically, authors have expressed concerns with respect to irreversibility of cardiac arrest, cannulation of the potential donor for the purpose of organ preservation without prior consent, possible re-establishment of oxygenated reperfusion of the brain after declaring death, and potential conflict of interests between resuscitation attempts and organ-preserving measures. Some authors have recommended a clarification of the abovementioned concerns before the further implementation of protocols for uDCD [6, 37, 41, 42, 44], whereas others have called for a moratoria in currently active protocols [43]. A bundle of novel therapies are in evolution for treating selected patients suffering from a refractory cardiac arrest (e.g., extracorporeal resuscitation and support, percutaneous coronary intervention, intra-aortic balloon pump, thrombolysis, and mild hypothermia, all deployed during or early after resuscitation attempts). Results, where this approach has been already implemented, are encouraging in terms of long-term survival with good neurologic recovery in some of these patients [54–63]. The availability of these interventions poses potential conflicts of interest between lifesaving and organ-preserving strategies [41, 42, 46]. Some of us [7, 51, 64, 65], and many other authors [38–42, 46, 48, 49, 53, 66], have suggested different approaches, seeking to save lives, when still feasible, but providing also the option of organ donation when all lifesaving clinical efforts have been exhausted. Thus, a joint venture between clinical and research communities in transplantation and resuscitation should combine both strategies in order to improve resuscitation outcomes while expanding uDCD.

This systematic review has several limitations. Although organisations provided us with draft protocols or guidelines for uDCD, only fully developed guidelines were included in the review, reducing the overall scope of guidelines to assess. The AGREE II appraisal process was used to assess the quality of guideline development. Though well supported, this tool is not the only accepted method for this purpose. The lack of homogenous data from the studies reporting transplant outcomes also precluded a meta-analysis and prevented the linking of outcomes to specific protocols used for the uDCD process.

## Conclusions

To the best of our knowledge, this is the first systematic review to compare the worldwide variability in practices, protocols, and transplant outcomes for uDCD in order to inform future protocol development and health policy. We conclude that uDCD is a viable option for increasing the organ donation pool. Despite variations in practice and heterogeneity of outcomes, uDCD yields success in kidney, liver, and lung transplantation. The implementation of uDCD has significant medical and logistic complexities, and international leaders should be recognized for their efforts. Depending on regional perspectives, there are a number of procedural, medical, legal, and ethical challenges that include definitions of refractory cardiac arrest, time limits for organ ischaemia, timing and type of consent required, determination of death, and organ-preserving interventions. Given the limited levels of evidence on which the current guidelines are based as well as the lack of both standardized definitions and processes between guidelines, it is not possible to recommend one protocol over another. Further standardization of guidelines and outcomes is required. Further research is required into the role of extracorporeal resuscitation and other novel therapies for treatment of refractory cardiac arrest of cardiac origin. The maintenance of trust by health professionals and by the public is recognized as a key point for the long-term success and widespread implementation of the valuable and promising uDCD strategy.

## Key messages

- The uDCD is a viable option for increasing the organ donation pool, yielding success in kidney, liver, and lung transplantation.
- Depending on regional perspectives, there are a number of procedural, medical, legal, and ethical challenges such as definitions of refractory cardiac arrest, time limits for organ ischaemia, timing and type of consent required, determination of death, and organ-preserving interventions.
- Current guidelines for uDCD are based on limited levels of evidence
- Standardization of definitions and processes would avoid the current existing variability in practices and heterogeneity of outcomes
- The maintenance of trust by health professionals and by the public is a key point for the long-term success and widespread implementation of the uDCD strategy.

## Additional files

Additional file 1:
**Guideline assessment with AGREE II (Appraisal of Guidelines for Research and Evaluation II) tool.**
Additional file 2:
**Quality assessment with Downs and Black scale.**
Additional file 3:
**Outcomes of included studies.**

## Abbreviations

AGREE: Appraisal of Guidelines for Research and Evaluation
cDCD: Controlled donation after circulatory death
CPR: Cardiopulmonary resuscitation
DCD: Donation after circulatory death
ECMO: Extracorporeal membrane oxygenation
h-ECMO: Hypothermic extracorporeal membrane oxygenation
IHCA: In-hospital cardiac arrest
n-ECMO: Normothermic extracorporeal membrane oxygenation
OHCA: Out-of hospital cardiac arrest
uDCD: Uncontrolled donation after circulatory death
WLST: Withdrawal of life-sustaining therapies

## References

[1] Matesanz R, Mahillo B, Alvarez Mar Carmona M. International figures on donation and transplantation - 2013. In: Newsletter Transplant. Spain: Organización Nacional de Trasplantes (ONT); 2014.

[2] Canadian Organ Replacement Register Annual Report. Treatment of End-Stage Organ Failure in Canada, 2004 to 2013. 2015. https://secure.cihi.ca/estore/productFamily.htm?locale=enandpf=PFC2864andlang=en. Accessed 16 June 2015.

[3] Borry P, van Reusel W, Roels L, Schotsmans P (2008). Donation after Uncontrolled Cardiac Death (uDCD): a review of the debate from a European perspective. J Law Med Ethics.

[4] Wall SP, Kaufman BJ, Gilbert AJ, Yushkov Y, Goldstein M, Rivera JE (2011). Derivation of the uncontrolled donation after circulatory determination of death protocol for New York city. Am J Transplant.

[5] Blackstock MJ, Ray DC (2014). Organ donation after circulatory death: an update. Eur J Emerg Med.

[6] Childress JF (2008). Organ donation after circulatory determination of death: lessons and unresolved controversies. J Law Med Ethics.

[7] Rodriguez-Arias D, Deballon IO (2012). Protocols for uncontrolled donation after circulatory death. Lancet.

[8] Morrissey PE, Monaco AP (2014). Donation after circulatory death: current practices, ongoing challenges, and potential improvements. Transplantation.

[9] Domínguez-Gil B, Haase-Kromwijk B, Van Leiden H, Neuberger J, Coene L, Morel P (2011). Current situation of donation after circulatory death in European countries. Transpl Int.

[10] Centre for Reviews and Dissemination (CRD) (2009). Systematic Reviews: CRD’s guidance for undertaking systematic reviews in health care.

[11] Downs SH, Black N (1998). The feasibility of creating a checklist for the assessment of the methodological quality both of randomised and non-randomised studies of health care interventions. J Epidemiol Community Health.

[12] Brouwers MC, Kho ME, Browman GP, Burgers JS, Cluzeau F, Feder G (2010). AGREE II: advancing guideline development, reporting and evaluation in health care. CMAJ.

[13] Gámez P, Córdoba M, Ussetti P, Carreño MC, Alfageme F, Madrigal L (2005). Lung transplantation from out-of-hospital non-heart-beating lung donors. one-year experience and results. J Heart Lung Transplant.

[14] Gagandeep S, Matsuoka L, Mateo R, Cho YW, Genyk Y, Sher L (2006). Expanding the donor kidney pool: utility of renal allografts procured in a setting of uncontrolled cardiac death. J Heart Lung Transplant.

[15] Sánchez-Fructuoso AI, Marques M, Prats D, Conesa J, Calvo N, Pérez-Contín MJ (2006). Victims of cardiac arrest occurring outside the hospital: a source of transplantable kidneys. Ann Intern Med.

[16] Fondevila C, Hessheimer AJ, Ruiz A, Calatayud D, Ferrer J, Charco R (2007). Liver transplant using donors after unexpected cardiac death: novel preservation protocol and acceptance criteria. Am J Transplant.

[17] Suárez F, Otero A, Solla M, Arnal F, Lorenzo MJ, Marini M (2008). Biliary complications after liver transplantation from maastricht category-2 non-heart-beating donors. Transplantation.

[18] Fieux F, Losser MR, Bourgeois E, Bonnet F, Marie O, Gaudez F (2009). Kidney retrieval after sudden out of hospital refractory cardiac arrest: a cohort of uncontrolled non heart beating donors. Crit Care.

[19] Gómez-Gutiérrez M, Aguirrezabalaga J, Quintela J, Suárez F. Uncontrolled donors after cardiac arrest or Maastricht type I and II donors. Outcomes. Resultados Medicina Clínica. 2009;10:24–5.

[20] Jiménez-Galanes S, Meneu-Diaz MJ, Elola-Olaso AM, Pérez-Saborido B, Yiliam FS, Calvo AG (2009). Liver transplantation using uncontrolled non-heart-beating donors under normothermic extracorporeal membrane oxygenation. Liver Transpl.

[21] Mateos-Rodríguez AA, Vázquez JC, Pascual JN, et al. An analysis after four year running a prehospital non-heart beating donor program. Emergencias. 2010;22:96–100.

[22] Mateos-Rodriguez A, Pardillos-Ferrer L, Navalpotro-Pascual JM, Barba-Alonso C, Martin-Maldonado ME, Andres-Belmonte A (2010). Kidney transplant function using organs from non-heart-beating donors maintained by mechanical chest compressions. Resuscitation.

[23] Hoogland ER, Snoeijs MG, Winkens B, Christaans MH, van Heurn LW (2011). Kidney transplantation from donors after cardiac death: uncontrolled versus controlled donation. Am J Transplant.

[24] Rodríguez DA, Del Río F, Fuentes ME, Naranjo S, Moradiellos J, Gómez D (2011). Lung transplantation with uncontrolled non-heart-beating donors. Transplantation. Donor prognostic factor and immediate evolution post transplant. Arch Bronconeumol.

[25] Fondevila C, Hessheimer AJ, Flores E, Ruiz A, Mestres N, Calatayud D (2012). Applicability and results of Maastricht type 2 donation after cardiac death liver transplantation. Am J Transplant.

[26] Gomez-de-Antonio D, Campo-Cañaveral JL, Crowley S, Valdivia D, Cordoba M, Moradiellos J (2012). Clinical lung transplantation from uncontrolled non-heart-beating donors revisited. J Heart Lung Transplant.

[27] Hanf W, Codas R, Meas-Yedid V, Berthiller J, Buron F, Chauvet C (2012). Kidney graft outcome and quality (after transplantation) from uncontrolled deceased donors after cardiac arrest. Am J Transplant.

[28] Reznik ON, Skvortsov AE, Reznik AO, Ananyev AN, Tutin AP, Kuzmin DO (2013). Uncontrolled donors with controlled reperfusion after sixty minutes of asystole: a novel reliable resource for kidney transplantation. PLoS One.

[29] Ribalta A, Gallardo J, Ruiz A, Deulofeu R. Donation after cardiac arrest program in Catalonia (Spain). Med Clin. 2009;10:18–21.

[30] Agence de la biomédecine. Antoine C, Tenaillon A. Exigible conditions for running a NHBD program for kidney procurement. Avril 2007. http://www.urgences-serveur.fr/IMG/pdf/DVprotocole_V12_avril_2007.pdf. Accessed 16 June 2015.

[31] Matesanz Acedos R, Coll Torres E, Domínguez-Gi González B, Perojo Vega L. Donacion en Asistolia en Espana: situacion actual y recomendaciones. ONT. 2012. http://www.ont.es/infesp/DocumentosDeConsenso/DONACI%C3%93N%20EN%20ASISTOLIA%20EN%20ESPA%C3%91A.%20SITUACI%C3%93N%20ACTUAL%20Y%20RECOMENDACIONES.pdf. Accessed 16 June 2015.

[32] Wälchli-Bhend S, Beyeler F. Programme DCD (Donor after Cardiac Death) en Suisse. Swisstransplant. Geneva, Switzerland. 2011.

[33] Currie I, Oniscu G, Reed M, Clegg G, Mackeown D, Forsythe J. Organ donation from the emergency department: category II DCD pilot programme policy document. UK Transplant. London, United Kingdom. 2013.

[34] Geraci PM, Sepe V (2011). Non-heart-beating organ donation in Italy. Minerva Anestesiol.

[35] Simon JR, Schears RM, Padela AI (2014). Donation after cardiac death and the emergency department: ethical issues. Acad Emerg Med.

[36] Roberts KJ, Bramhall S, Mayer D, Muiesan P (2011). Uncontrolled organ donation following prehospital cardiac arrest: a potential solution to the shortage of organ donors in the United Kingdom?. Transpl Int.

[37] Bell MD (2006). Emergency medicine, organ donation and the Human Tissue Act. Emerg Med J.

[38] Reed MJ, Lua SB (2014). Uncontrolled organ donation after circulatory death: potential donors in the emergency department. Emerg Med J.

[39] Manara AR, Thomas I (2010). The use of circulatory criteria to diagnose death after unsuccessful cardiopulmonary resuscitation. Resuscitation.

[40] Rudge C, Matesanz R, Delmonico FL, Chapman J (2012). International practices of organ donation. Br J Anaesth.

[41] Bracco D, Noiseux N, Hemmerling TM (2007). The thin line between life and death. Intensive Care Med.

[42] Doig CJ, Zygun DA (2008). (Uncontrolled) donation after cardiac determination of death: a note of caution. J Law Med Ethics.

[43] Joffe AR, Carcillo J, Anton N, de Caen A, Han YY, Bell MJ (2011). Donation after cardiocirculatory death: a call for a moratorium pending full public disclosure and fully informed consent. Philos Ethics Humanit Med.

[44] Dubois JM, Volpe RL (2008). Introduction: organ donation and death from unexpected circulatory arrest: engaging the recommendations of the Institute of Medicine. J Law Med Ethics.

[45] Rady MY, Verheijde JL, Johnstone MJ (2012). The general public is ready for transparency about organ donation at the end of life. Emerg Med J.

[46] Harrington MM (2009). The thin flat line: redefining who is legally dead in organ donation after cardiac death. Issues Law Med.

[47] Volk ML, Warren GJ, Anspach RR, Couper MP, Merion RM, Ubel PA (2010). Attitudes of the American public toward organ donation after uncontrolled (sudden) cardiac death. Am J Transplant.

[48] Zeiler K, Furberg E, Tufveson G, Welin S (2008). The ethics of non-heart-beating donation: how new technology can change the ethical landscape. J Med Ethics.

[49] Hanto DW, Veatch RM (2011). Uncontrolled donation after circulatory determination of death (UDCDD) and the definition of death. Am J Transplant.

[50] Caspers C, Zink B (2011). Organ preservation: a new frontier in emergency medicine. Acad Emerg Med.

[51] Rodriguez-Arias D, Ortega-Deballon I, Smith MJ, Youngner SJ (2013). Casting light and doubt on uncontrolled DCDD protocols. Hastings Cent Rep.

[52] Thuong M. Next of kin approach and information to be delivered during the process of donation after uncontrolled circulatory death. Ann Fr Med Urgence. 2011;1:438–41.

[53] Goudet V, Albouy-Llaty M, Migeot V, Pain B, Dayhot-Fizelier C, Pinsard M (2013). Does uncontrolled cardiac death for organ donation raise ethical questions? An opinion survey. Acta Anaesthesiol Scand.

[54] Jaski BE, Ortiz B, Alla KR, Smith SC, Glaser D, Walsh C (2010). A 20-year experience with urgent percutaneous cardiopulmonary bypass for salvage of potential survivors of refractory cardiovascular collapse. J Thorac Cardiovasc Surg.

[55] Gaieski DF, Boller M, Becker LB (2012). Emergency cardiopulmonary bypass: a promising rescue strategy for refractory cardiac arrest. Crit Care Clin.

[56] Reynolds JC, Frisch A, Rittenberger JC, Callaway CW (2013). Duration of resuscitation efforts and functional outcome after out-of-hospital cardiac arrest: when should we change to novel therapies?. Circulation.

[57] Wallmüller C, Sterz F, Testori C, Schober A, Stratil P, Hörburger D (2013). Emergency cardio-pulmonary bypass in cardiac arrest: seventeen years of experience. Resuscitation.

[58] Stub D, Bernard S, Pellegrino V, Smith K, Walker T, Sheldrake J (2015). Refractory cardiac arrest treated with mechanical CPR, hypothermia, ECMO and early reperfusion (the CHEER trial). Resuscitation.

[59] Greenwood JC, Herr DL (2014). Mechanical circulatory support. Emerg Med Clin North Am.

[60] Massetti M, Gaudino M, De Paulis S, Scapigliati A, Cavaliere F (2014). Extracorporeal membrane oxygenation for resuscitation and cardiac arrest management. Heart Fail Clin.

[61] Mochizuki K, Imamura H, Iwashita T, Okamoto K (2014). Neurological outcomes after extracorporeal cardiopulmonary resuscitation in patients with out-of-hospital cardiac arrest: a retrospective observational study in a rural tertiary care center. J Intensive Care.

[62] Johnson NJ, Acker M, Hsu CH, Desai N, Vallabhajosyula P, Lazar S (2014). Extracorporeal life support as rescue strategy for out-of-hospital and emergency department cardiac arrest. Resuscitation.

[63] Wang CH, Chou NK, Becker LB, Lin JW, Yu HY, Chi NH (2014). Improved outcome of extracorporeal cardiopulmonary resuscitation for out-of-hospital cardiac arrest–a comparison with that for extracorporeal rescue for in-hospital cardiac arrest. Resuscitation.

[64] Ortega-Deballon I, De la Plaza-Horche E (2014). Protocols for uncontrolled donation after circulatory death: a comprehensive approach to refractory cardiac arrest. Acad Emerg Med.

[65] Ortega-Deballon I, Vailhen DR, Smith MJ (2013). Uncontrolled donation after circulatory determination of death protocols: ethical challenges and suggestions for improvement. Ann Emerg Med.

[66] Hoogland ER, Snoeijs MG, van Heurn LW (2010). DCD kidney transplantation: results and measures to improve outcome. Curr Opin Organ Transplant.

