# Supplementary material for: Protocols for uncontrolled donation after circulatory death: a systematic review of international guidelines, practices and transplant outcomes
Source: Crit Care. 2015 Jun 24;19(1):268. doi: 10.1186/s13054-015-0985-7 (PMC4495857; doi:10.1186/s13054-015-0985-7)
Supplement: Additional file 1: — Guideline assessment with AGREE II (Appraisal of Guidelines for Research and Evaluation II) tool. [file 13054_2015_985_MOESM1_ESM.docx]

| **Appendix 1:** Appraisal of Guidelines by AGREE II | | | | | | |
| --- | --- | --- | --- | --- | --- | --- |
|  | QUALITY SCORES* | | | | | |
| National/Regional Guideline  Country; region (year) | Domain 1  Scope and  Purpose | Domain 2  Stakeholder Involvement | Domain 3  Rigour of Development | Domain 4  Clarity of Presentation | Domain 5  Applicability | Domain 6  Editorial Independence |
| **FRANCE (2007)^30^** | 69% | 39% | 9% | 67% | 19% | 6% |
| **ITALY; Pavia (2011)^34^** | 72% | 39% | 13% | 74% | 40% | 17% |
| **SWITZERLAND (2011)^32^** | 43% | 24% | 9% | 80% | 26% | 0% |
| **US; New York City (2011)^4^** | 96% | 81% | 51% | 74% | 60% | 69% |
| **SPAIN (2012)^31^**  Alicante, Barcelona, Castilla La Mancha  Granada, Galicia, Madrid City & Region | 85% | 43% | 12% | 74% | 28% | 8% |
| **UNITED KINGDOM; Scotland (2013)^33^** | 93% | 57% | 24% | 83% | 51% | 22% |
| *Quality Scores were calculated as follows:  Scaled domain score = (Obtained score – Minimum possible score) / (Maximum possible score – Minimum possible)  Maximum possible score = 7 (strongly agree) x (# items in domain) x (# appraisers)  Minimum possible score = 1 (strongly disagree) x (# items in domain) x (# appraisers)  3 appraisers reviewed all documents. | | | | | | |
