# Supplementary material for: Protocols for uncontrolled donation after circulatory death: a systematic review of international guidelines, practices and transplant outcomes
Source: Crit Care. 2015 Jun 24;19(1):268. doi: 10.1186/s13054-015-0985-7 (PMC4495857; doi:10.1186/s13054-015-0985-7)
Supplement: Additional file 2: — Quality assessment with Downs and Black scale. [file 13054_2015_985_MOESM2_ESM.docx]

| **Appendix 2:** Risk of bias and methodological quality assessment* of included outcome studies | | | | |
| --- | --- | --- | --- | --- |
| Study | Reporting | External | Internal Validity | |
|  | (/11) | Validity (/3) | Bias  (/7) | Confounding  (/6) |
| Gámez 2005^13^ | 6 | 0 | 4 | 2 |
| Gagandeep 2006^14^ | 10 | 2 | 4 | 3 |
| Sánchez-Fructuoso 2006^15^ | 11 | 0 | 6 | 0 |
| Fondevila 2007^16^ | 7 | 0 | 6 | 2 |
| Suárez 2008^17^ | 11 | 0 | 4 | 2 |
| Fieux 2009^18^ | 8 | 1 | 5 | 2 |
| Gómez Gutiérrez 2009^19^ | 6 | 0 | 3 | 2 |
| Jiménez-Galanes 2009^20^ | 9 | 0 | 5 | 3 |
| Mateos-Rodríguez 2010^21^ | 5 | 1 | 1 | 3 |
| Mateos-Rodríguez 2010^22^ | 9 | 1 | 4 | 4 |
| Geraci 2011^34^ | 6 | 1 | 4 | 2 |
| Hoogland 2011^23^ | 10 | 2 | 5 | 2 |
| Rodríguez 2011^24^ | 10 | 2 | 5 | 3 |
| Fondevila 2012^25^ | 8 | 0 | 5 | 3 |
| Gómez-de-Antonio 2012^26^ | 10 | 0 | 5 | 3 |
| Hanf 2012^27^ | 10 | 0 | 5 | 3 |
| Reznick 2013^28^ | 10 | 0 | 5 | 3 |
| *Assessment performed using a modified version of Downs and Black checklist | | | | |
