# Supplementary material for: Protocols for uncontrolled donation after circulatory death: a systematic review of international guidelines, practices and transplant outcomes
Source: Crit Care. 2015 Jun 24;19(1):268. doi: 10.1186/s13054-015-0985-7 (PMC4495857; doi:10.1186/s13054-015-0985-7)
Supplement: Additional file 3: — Outcomes of included studies. [file 13054_2015_985_MOESM3_ESM.docx]

**Appendix 3:** Outcomes of Included Studies

| Study | Organ | Donor Sample Size | | | | Main outcomes | Results | | | p value | |
| --- | --- | --- | --- | --- | --- | --- | --- | --- | --- | --- | --- |
|  |  | uDCD | cDCD | | NDD |  | uDCD | cDCD | NDD |  |  |
|  |  |  |  | |  |  |  |  |  |  | |
| Gámez 2005^13^ | Lung | 5 | - | | - | n= 5 Tx, 4 BL, 1 UL  Complications, n (%)  Time to extubation, range  Hospital stay, range  All survived to publication date -time range | 3 (60)  21h-37d  20d-66d  2m-13m | - | - | - | |
| Rodríguez 2011^24^ | Lung  32 of 331 p | 32 | - | | - | n=33 , 19 BL, 13 UL Tx, 1 died in operating room  Time to extubation, median, range  Hospital stay, median, range  Primary graft dysfunction (%)  1-Mo Mortality (%) | 48h  36d-144d  34.5 26.5d-59.7d  46.9  12.1 | -  -  -  - | -  -  -  - | -  -  -  - | |
| Gomez-de-Antonio 2012^26^ | Lung | 29 | - | | - | 29 Tx, 18 BL, 11 UL  Primary graft dysfunction G1 (%)  Primary graft dysfunction G2 (%)  Primary graft dysfunction G3 (%)  3-Mo Patient survival (%)  1-Year Patient survival (%)  3-Year Patient survival (%)  5-Year Patient survival (%) | 17  17  38  78  68  57  51 | -  -  -  -  -  -  - | -  -  -  -  -  -  - | -  -  -  -  -  -  - | |
| Gagandeep 2006^14^ | Kidney | 216 | 1814 | 75 865 | | Graft survival (%)  Patient survival (%)  Primary graft non-function (%)  Delayed graft function (%) | NNR  NNR  NNR  51 | NNR  NNR  NNR  42 | NNR  NNR  NNR  24 | 0.65 uDCD*  0.89 cDCD*  0.99 uDCD*  0.70 cDCD*  0.10 uDCD*  0.17 cDCD*  <0.001 uDCD*  <0.001 cDCD* | |
|  |  |  |  | |  |  |  |  |  | |  |
| Sánchez-Fructuoso 2006^15^ | Kidney | 320 | - | | 458# | n=320 uDCD, n=458 NDD Tx  Non-viable transplantations (%)  Delayed graft function (%)  1-Year Graft survival (%)  5-Year Graft survival (%)  1-Year Patient survival (%)  5-Year Patient survival (%) | 4.4  60.9  87.4  82.1  95  90 | -  -  -  - | 1.1  20.4  90.7  85.5  97  91 | | 0.01  < 0.001  0.22  -  0.22  - |
| Fieux 2009^18^ | Kidney  (27 of 122 p) | 27 | - | | - | n=31 Tx, 24 included in study  Delayed graft function (%)  Creatinine clearance at 6 mo (mean±SD, ml/min, n=22)  6-Mo Graft survival (%) | 92  66±24  89 | -  -  - | -  -  - | | -  -  - |
| Mateos-Rodríguez 2010^22^ | Kidney  20 MCC  28 MeCC | 48 |  | |  | Kidneys transplanted (% of p)  MCC (n=33 Tx)  MeCC (n=39 Tx) | 82  70 | -  - | -  - | | 0.23 |
|  |  |  |  | |  | Primary graft failure (%)  MCC  MeCC | 9.1  5.1 | -  - | -  - | | 0.50 |
| Geraci 2011^34^ | Kidney  7 donors provided 7 kidneys | 7 | - | | - | n= 7  Primary graft failure (n)  Delayed graft function, range  Creatinine at last follow-up, range in mg/dl | 1  2d-19d  1.3-1.8 | -  -  - | -  -  - | | -  -  -  - |
|  |  |  |  | |  |  |  |  |  | |  |
| Hoogland 2011^23^ | Kidney | 83 | 111 | | - | n=128 uDCD Tx; n=208 cDCD Tx  Kidneys transplanted (% of p)  Primary graft non-function (%)  Delayed graft function (%)  5-Year Graft survival (%)  10-Year Graft survival (%)  5-Year Patient survival (%)  10-Year Patient survival (%) | 52  22  61  63  50  78  61 | 87  21  56  64  46  80  60 | -  -  -  -  -  -  - | | <0.001  0.43  0.43  0.74  0.74  0.76  0.76 |
| Hanf 2012^27^** | Kidney | 27 |  | |  | n=27  Primary graft non-function (%)  Delayed graft function (%)  1-Year Graft survival (%)  3-Year Graft survival (%)  1-Year Patient survival (%)  3-Year Patient survival (%) | 0  81.5  100  100  100  100 |  |  | |  |
|  |  |  |  | |  |  |  |  |  | |  |
| Reznick 2013^28^ | Kidney | 22 | - | | 92 | n= 44 uDCD Tx; n= 92 NDD Tx  Primary graft non-function (%)  Delayed graft function (%)  Early acute rejection, 3 mo (%)  Late acute rejection, 12 mo (%)  Surgical complications (%)  1-Year Creatinine, mean±SD (mg/dl)  1-Year Graft survival (%) | 0  52.3  4.6  4.5  2.5  0.116  ±0.004  95.5  (n=42) | -  -  -  -  -  -  - | 0  36.9  9.8  4.4  2.2  0.116  ±0.004  94.6  (n=87) | | -  -  -  -  -  >0.05  -  - |
|  |  |  |  | |  |  |  |  |  | |  |
| Fondevila 2007^16^ | Liver | 10  of 40p | - | | 20^ | n= 10 uDCD Tx; n= 20 NDDTx  Primary graft non-function (%)  Hepatic artery thrombosis (%)  1-Year Graft survival (%)  1-Year Patient survival (%) | 10  10  50  70 | -  -  -  - | 0  5  75  80 | | -  -  NSD  NSD |
| Suárez 2008^17^ | Liver | 27 | - | | 471 | n= 27 uDCD Tx; n= 471 NDD Tx  Any type of biliary complication  Nonanastomotic biliary strictures  Primary non-function (%)  5-Year Graft survival (%)  5-Year Patient survival (%) | 41.7  NNR  18  49  62 | -  -  -  -  - | 16.8  NNR  3  68  74 | | 0.66  <0.001  <0.001  <0.001  0.229 |
|  |  |  |  | |  |  |  |  |  | |  |
| Gómez Gutierrez 2009 ^19^ | Liver  CHUAC  14 of 29 p  HCSC  17 of 26 p | 31 | - | | 471 | n=31 uDCD Tx; n=471 NDD Tx  Primary graft non-function (%)  Any type of biliary complication (%)  Non-anastomotic stenosis (%)  5-Year Graft survival (%)  5-Year Patient survival (%) | 18  41.7  25  49  62 | -  -  -  -  - | 3  16.8  2.3  68  74 | | <0.001  NSD  <0.001  <0.001  <0.001  NSD |
| Jiménez-Galanes 2009^20^ | Liver  20 of 43 who began procedure | 20 | - | | 40~ | n=20 uDCD Tx; n=40 NDD Tx  Incidence of ischemic Cholangiopathy (%)  Primary graft non-function (%)  1-Year Graft survival (%)  1-Year Patient survival (%) | 5  10  80  85.5 | -  -  -  - | 0  2.5  87.5  87.5 | | 0.15  0.21  0.774  0.768 |
| Fondevila 2012^25^ | Liver  34 of 400 p | 34 | - | | - | n=34 Tx  Liver turned down due to Absolute contraindications (%)  Liver turned down due to relative contraindications (%)  1-Year Graft survival (%)  1-Year Patient survival (%) | 59  32  70  82 | -  -  -  - | -  -  -  - | | -  -  -  - |
| Ribalta 2009^29^ | Kidney  Liver  (21 donors) | 34  4 | -  - | | -  - | No outcomes reported  No outcomes reported | -  - | -  - | -  - | | -  - |
|  |  |  |  | |  |  |  |  |  | |  |
| Mateos-Rodríguez 2010^21^ | Kidney,  Liver &  Lung  (82 of 132 p) | 82 | - | | - | n=158 Kidney, n=16 Liver, n=13 Lung Tx  Primary graft non-function-Kidney (%)  Rejections – Kidney (%)  Acute rejection– Liver (%) | 9  9  25 | -  -  - | -  -  - | | -  -  - |

Legend

Tx – transplants; BL – bilateral lung transplant; UL – unilateral lung transplant

uDCD – Donor from uncontrolled donation after cardiac death procedure

cDCD – Donor from controlled donation after cardiac death procedure

NDD – Donor from donation after neurological determination of death procedure

NNR – Exact number not reported

NSD – Reported as “not significantly different. No p value given

* - NDD was used as the reference for comparison

# - NDD age <60

^ - A 2:1 matching of controls for age, sex, Model for End-stage Liver Disease (MELD) score, and body mass index (BMI)

~ - A 2:1 matching of controls. Matching criteria not disclosed

p – Potential donors

CHUAC – Centro Hospitalario Universitario A Coruña, Spain

HCSC – Hospital Clinico San Carlos de Madrid, Spain

BO - Bronchiolitis obliterans

MCC – Donor was maintained with manual chest compressions; MeCC – Donor was maintained with mechanical chest compression device

pr – Procured

G1 - Grade 1, G2 – Grade 2, G3 – Grade 3

** Study by Hanff 2012 did not use cDCD nor NDD as comparison groups but rather extended criteria donors (ECD, n= 30) and simultaneous pancreas kidney (SPK, n=24) donors. There were no cases of primary graft non-function for either of these two groups. There were no cases of delayed graft function in the SPK group but the incidence was 27.6% for the ECD group, which was significantly lower (p<0.001) compared to the uDCD group. 1-year graft survival for the SPK group was 100% but it was 90% in the ECD group, significantly lower compared to the uDCD group. 3-year graft survival was 82% for the ECD and 94% for SPK while the 1 and 3–year patient survivals were 100% for both groups.
